# Acantholysis may precede elevation of circulating anti‐desmoglein 3 antibody levels in pemphigus vulgaris presenting with desquamative gingivitis

**DOI:** 10.1002/cre2.174

**Published:** 2019-03-04

**Authors:** Hiroyasu Endo, Terry D. Rees, Hideo Niwa, Kayo Kuyama, Maya Oshima, Tae Serizawa, Shigeo Tanaka, Masamichi Komiya, Takanori Ito

**Affiliations:** ^1^ Department of Oral Diagnosis Nihon University School of Dentistry at Matsudo Matsudo Japan; ^2^ Department of Periodontics Texas A&M College of Dentistry Dallas Texas USA; ^3^ Department of Head and Neck Surgery Nihon University School of Dentistry at Matsudo Matsudo Japan; ^4^ Department of Pathology Nihon University School of Dentistry at Matsudo Matsudo Japan; ^5^ Department of Oral Surgery Nihon University School of Dentistry at Matsudo Matsudo Japan

**Keywords:** autoimmune diseases, desmoglein 3, gingival diseases, pemphigus

## Abstract

Pemphigus vulgaris (PV) is an autoimmune, blistering disease that affects the mucosa and skin. The current theory favors the concept that anti‐desmoglein (Dsg) 3 autoimmunity is the only pathogenic event needed to induce acantholysis. However, a few cases of active PV in the oral cavity had no detectable anti‐Dsg 3 antibody. The aim of this study was to evaluate the differences in clinical and laboratory findings, whether or not the anti‐Dsg 3 antibodies were present. This study was based on a retrospective review of 10 PV cases. The evaluation of the circulating autoantibody titers to Dsg 3 was conducted by using enzyme‐linked immunosorbent assay (ELISA). An index value of 20 or more was used as the cutoff for a positive reaction. Only five of the 10 PV cases had a positive Dsg 3 ELISA. There were no differences in clinical, cytological, histopathological, and direct immunofluorescence findings, whether or not the anti‐Dsg 3 antibodies were present. Of the five patients with a negative reaction at the time of diagnosis, the Dsg 3 ELISA became positive in the follow‐up period in three cases. In the remaining two cases, the Dsg 3 ELISA was consistently negative for 18 months. Dsg 3 ELISA was negative early in some PV cases. Therefore, PV acantholysis may precede the elevation of circulating anti‐Dsg 3 antibody levels. The diagnosis of PV should be considered based on comprehensive clinical, histopathological, and immunofluorescent criteria.

## 1 INTRODUCTION

Pemphigus vulgaris (PV) is an autoimmune blistering disease that affects mucous membranes and the skin. Although it rarely occurs, PV is a life‐threatening condition if left untreated, so it is important to diagnose and treat it in its early stages. Oral lesions are the most common early evidence of the disease and will ultimately develop in almost all patients with untreated PV (Endo et al., 2005; Endo, Rees, Hallmon, et al., 2008; Endo & Rees, 2011). Occasionally, the gingiva is the only site involved in the early lesions, and desquamative gingivitis is a relatively common manifestation of the disease (Endo et al., 2005; Endo & Rees, 2011; Endo, Rees, Hallmon, et al., 2008). Gingival Nikolsky's sign showed a positive reaction in more than 90% of the cases that were diagnosed as PV based on oral lesions (Mignogna et al., 2008). This phenomenon can be induced by the application of a shearing force on normal‐appearing gingiva, producing epithelial desquamation. Histopathologically, PV is characterized by acantholysis and a suprabasilar split in the epithelium. Acantholytic (Tzanck) cells are often found in intraepithelial clefts in a hematoxylin–eosin (H&E)‐stained section. A presumptive diagnosis of PV can be established by confirming the presence of Tzanck cells in the cytologic smear obtained by scraping the gingival lesions (Endo, Rees, Kuyama, et al., 2008).

During various phases of the disease, PV patients may have autoantibodies to various antigens on the keratinocyte surface in the oral mucosa, skin tissue, and serum. These antibodies are often detected during direct immunofluorescence (DIF) and indirect immunofluorescence examinations (Avgerinou et al., 2013; Endo et al., 2005; Endo, Rees, Hallmon, et al., 2008; Lamey et al., 1992; Zagorodniuk et al., 2005). There is strong evidence to support the notion that the PV autoantibodies are pathogenic, and they can cause acantholysis in the epithelium (Anhalt, Labib, Voorhees, Beals, & Diaz, 1982). Although it has been determined that the principal autoantigens in pemphigus patients are desmogleins (Dsgs), which are the components of desmosomes in the epidermis and mucous membranes, PV pathogenesis is still under extensive debate (Ahmed et al., 2016; Amagai et al., 2006; Amagai, Klaus‐Kovtun, & Stanley, 1991; Amagai, Tsunoda, Zillikens, Nagai, & Nishikawa, 1999; Amber, Valdebran, & Grando, 2018; Cirillo, Cozzani, Carrozzo, & Grando, 2012; Di Zenzo, Borradori, & Muller, 2017; Grando, 2012; Sardana, Garg, & Agarwal, 2013; Stanley, Nishikawa, Diaz, & Amagai, 2001). Detection of anti‐Dsg 3 and anti‐Dsg 1 autoantibodies using the enzyme‐linked immunosorbent assay (ELISA) test is widely used in the serologic diagnosis of pemphigus. Almost all patients with PV lesions restricted to the oral mucosa have only anti‐Dsg 3 antibody in the serum, whereas patients with an advanced case of the disease involving the oral mucosa and skin may have both anti‐Dsg 3 and anti‐Dsg 1 antibodies (Amagai et al., 1999; Endo et al., 2005; Endo, Rees, Hallmon, et al., 2008). Pemphigus foliaceus (PF) is a somewhat less severe form of pemphigus that affects superficial skin and very rarely the oral mucosa. Serum from patients with PF contains only anti‐Dsg 1 autoantibodies (Amagai et al., 1999; Zagorodniuk et al., 2005). Although the ELISA test is a sensitive tool for the diagnosis of pemphigus, antibody titers do not always relate to the disease activity. Available data indicate that a few patients with PV may have no detectable anti‐Dsg 3 antibody, even though they have active disease in the oral cavity (Avgerinou et al., 2013; Belloni‐Fortinaet al., 2009; Daneshpazhooh et al., 2007; Jamora, Jiao, & Bystryn, 2003; Khandpur, Sharma, Sharma, Pathria, & Satyam, 2010; Koga et al., 2012; Sharma, Prasad, Khandpur, & Kumar, 2006; Zagorodniuk et al., 2005). The current theory about pemphigus pathogenesis favors the idea that anti‐Dsg 3 autoimmunity is the necessary and pathogenic event to cause acantholysis in PV patients with oral lesions (Amagai et al., 2006; Stanley et al., 2001). If that is the case, PV patients with active oral disease but no evidence of anti‐Dsg 3 antibody would fall into a different PV category. It is not clear whether these PV patients have the same clinical or laboratory findings as PV patients having anti‐Dsg 3 antibodies. The aim of this study was to evaluate the differences in clinical, cytological, histopathological, and DIF features, based on the presence or absence of anti‐Dsg 3 antibodies.

## 2 MATERIALS AND METHODS

This study was based on a retrospective review of 10 clinical records that were classified as PV (one male and nine females, aged 24 to 73 years [mean age, 46.3 years]) at Nihon University, School of Dentistry at Matsudo, from 2002 through 2015. The protocol of this study was approved by an institutional review board (Ethics Committee Approval no. EC14‐011‐1). The files for these patients contained information such as clinical site involvements, the presence of Nikolsky's sign, cytology, biopsy and DIF reports, and circulating autoantibody titers to Dsgs. Indirect immunofluorescence study was not examined in the author's facility because Dsgs assays are available routinely and covered by national health insurance in Japan. The clinical disease activity in each case was scored using the pemphigus disease area index (PDAI) (Ormond et al., 2018; Shimizu et al., 2014). For the purpose of this study, only oral mucosal sites were scored, with a possible total of 90 as reported by Ormond et al. (2018). A positive gingival Nikolsky's sign described the extension of the erosion on the surrounding normal‐appearing tissue by rubbing the edge of the affected area with a periodontal probe or the ease of inducing erosion by rubbing apparently unaffected gingiva distant from the lesions (Mignogna et al., 2008). Cytologic smears were obtained by scratching the gingiva with a cytobrush at the time of diagnosis. After smearing the collected cells on a glass slide, they were fixed immediately using 95% ethyl alcohol. All the smears were stained according to the Papanicolaou method. The smears were evaluated by oral pathologists for the presence or absence of Tzanck cells, which have round, swollen nuclei with a fine chromatin. A biopsy was obtained that included perilesional tissue and was then submitted for routine histopathology. An additional biopsy was obtained from normal‐appearing tissue for the DIF study. H&E‐stained sections were evaluated by oral pathologists for diagnostic features. All the DIF tests were analyzed, and the results were reported by certified dermatopathologists. Evaluation of the circulating autoantibody titers to Dsg 3 and Dsg 1 were conducted using the ELISA test. An index value of 20 or more was used as the cutoff for a positive reaction for both Dsg3 and Dsg 1 antibodies, as recommended by Amagai et al. (1999) The Dsg ELISA measurements were done at the time of diagnosis and during the follow‐up period in some patients.

## 3 RESULTS

**Figure 1:**
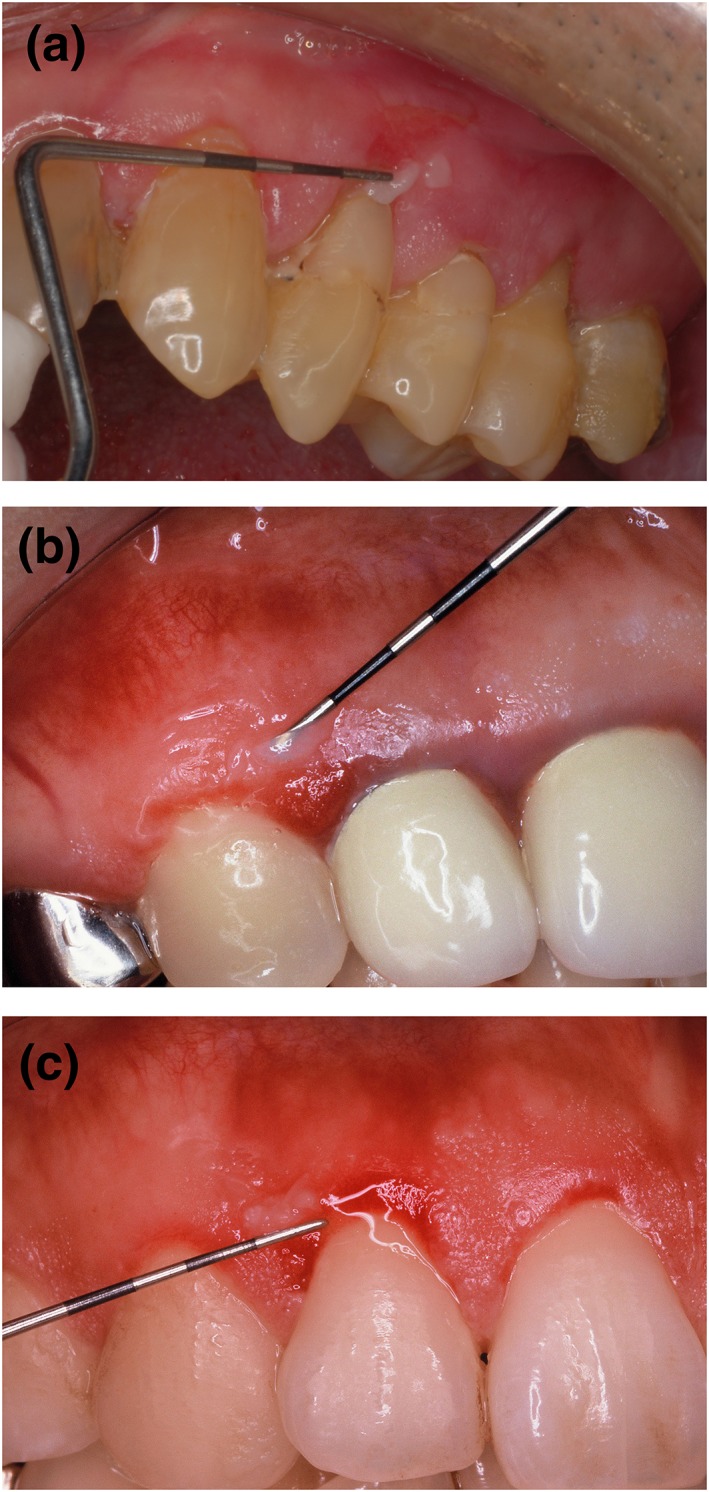
Desquamative lesions on the attached gingiva associated with pemphigus vulgaris. Nikolsky's sign was positive. (a) Case 1 with a high value of desmoglein (Dsg) 3 enzyme‐linked immunosorbent assay (ELISA). (b) Case 8 with a negative value of Dsg 3 ELISA. The Dsg 3 ELISA increased in the follow‐up period and became positive 9 months after diagnosis. (c) Case 9 with a negative Dsg 3 ELISA values. The Dsg 3 ELISA was consistently negative for 18 months after diagnosis

**Figure 2:**
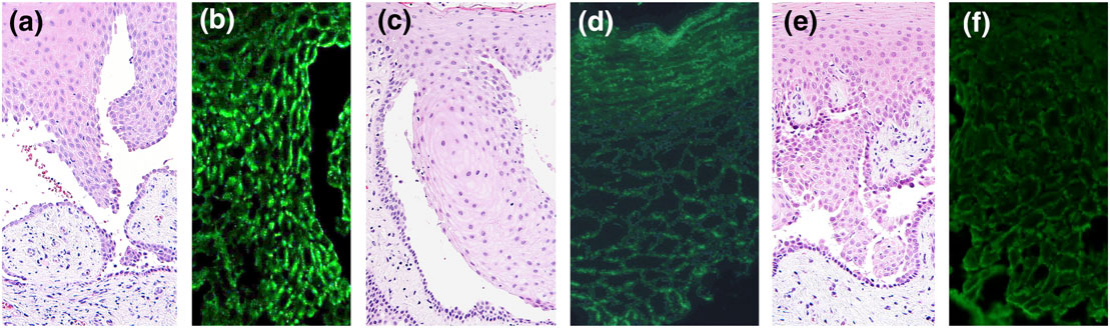
Histopathological and direct immunofluorescence features of pemphigus vulgaris (original magnification ×200). Hematoxylin–eosin‐stained section showing acantholysis and a suprabasilar split in Case 1 (a), Case 7 (c), and Case 10 (e). Direct immunofluorescence demonstrating intercellular deposition of immunoglobulin (Ig) G creating a “fishnet‐like” pattern in Case 1 (b), Case 7 (d), and Case 10 (f). Even if the desmoglein (Dsg) 3 enzyme‐linked immunosorbent assay (ELISA) had a high index value (Case 1) or the Dsg3 ELISA showed a negative reaction (Cases 7 and 10), acantholysis and a suprabasilar split and intercellular deposits of IgG were seen

The results of the clinical and laboratory findings of the 10 cases are shown in Tables 1 and 2. All the patients complained of painful, easily bleeding, erythematous gingiva. These clinical appearances were consistent with desquamative gingivitis. In addition, three patients (Cases 2, 4, and 6) had extragingival involvements (the buccal mucosa, tongue, or floor of mouth). The mean PDAI activity score for oral mucosa was 5.8 ranging from 2 to 18, and thus, the PV disease severity was mild to moderate. In all of the cases, positive Nikolsky's sign, acantholysis and a suprabasilar split in H&E‐stained sections, and intercellular deposits of immunoglobulin (Ig) G in DIF test were confirmed (Table 1 and Figures 1 and 2). The presence of Tzanck cells in the cytologic smears was also confirmed in all nine cases in which cytologic smears were obtained. In contrast, only five of the 10 cases (Cases 1–5) had a positive reaction, ranging from 28 to 426, in the Dsg 3 ELISA measurement at the time of diagnosis (Table 2). In the remaining five cases (Cases 6–10), the Dsg 3 ELISA results were very low and ranged between 5 and 19, indicating a negative reaction (Table 2). As a negative control, Dsg 3 ELISA measurements were also obtained from seven mucous membrane pemphigoid patients with desquamative gingivitis who had clinical, histopathological, and DIF studies consistent with that diagnosis. All seven patients showed a negative reaction, ranging from 5 to 6, at the time of diagnosis (data not shown).

**Table 1:** Summary of the clinical and laboratory findings of the 10 cases of pemphigus vulgaris

Case	Nikolsky's sign	Tzanck cells in cytologic smear	Suprabasilar split in H&E‐stained section	Intercellular deposits of IgG in DIF specimen	PDAI (oral mucosal sites)^a^
1	+	+	+	+	4
2	+	+	+	+	18
3	+	+	+	+	10
4	+	+	+	+	4
5	+	+	+	+	3
6	+	+	+	+	2
7	+	NP	+	+	7
8	+	+	+	+	4
9	+	+	+	+	2
10	+	+	+	+	4

*Note*. +: presence or positive; DIF: direct immunofluorescence; H&E: hematoxylin–eosin; Ig: immunoglobulin; NP: not performed; PDAI: pemphigus disease area index.

a Only oral mucosal sites were scored based on the number and size of the lesions with a possible total of 90. The sites include buccal mucosa, hard palate, soft palate, upper gingiva, lower gingiva, tongue, floor of mouth, labial mucosa, and posterior pharynx.

**Table 2:** Presence and level of Dsgs 3 and 1 ELISA of the 10 cases of pemphigus vulgaris

Case	Dsg 3 ELISA^a^ (index value)	Dsg 1 ELISA^a^ (index value)
1	426 (+)	7 (−)
2	320 (+)	5 (−)
3	125 (+)	43 (+)
4	56 (+)	5 (−)
5	28 (+)	NP
6	19 (−)	5 (−)
7	12 (−)	21 (+)
8	5 (−)	5 (−)
9	5 (−)	NP
10	5 (−)	6 (−)

*Note*. +: positive; −: negative; Dsg: desmoglein; ELISA: enzyme‐linked immunosorbent assay; NP: not performed.

a An index value of 20 or more was used as the cutoff for a positive reaction.

Dsg 1 ELISA measurement was conducted in eight of the 10 PV cases. Two of the eight cases (Cases 3 and 7) had a positive reaction at the time of diagnosis (Table 2).

Serial circulating autoantibody titers to Dsg 3 were conducted during the follow‐up period in five patients (Table 3). Of the five patients who had a negative reaction at the time of diagnosis, the Dsg 3 ELISA increased in the follow‐up period and became positive in three cases (Cases 6–8). In the remaining two cases (Cases 9 and 10), the Dsg 3 ELISA was consistently negative for 18 months after diagnosis.

**Table 3:** Serial circulating Dsg 3 ELISA levels during the follow‐up period in five patients

Case	At the time of diagnosis	Follow‐up period (months)				
		3	6	9	12	18
6	19 (−)	NP	52 (+)	NP	101 (+)	128 (+)
7	12 (−)	24 (+)	NP	7 (−)	NP	40 (+)
8	5 (−)	5 (−)	NP	36 (+)	110 (+)	129 (+)
9	5 (−)	5 (−)	NP	5 (−)	NP	5 (−)
10	5 (−)	5 (−)	5 (−)	NP	NP	5 (−)

*Note*. The first Dsg 3 ELISA measurement was made at the time of diagnosis. An index value of 20 or more was used as the cutoff for a positive reaction. None of the cases received systemic treatment during the follow‐up period. +: positive; −: negative; Dsg: desmoglein; ELISA: enzyme‐linked immunosorbent assay; NP: not performed.

After diagnosis of PV in the dental clinic, the patients were referred to dermatologists and skin lesions were confirmed in two cases (Cases 2 and 3). Because skin lesions were absent or minor, all the patients visited the dental clinic first. Two patients later developed skin lesions, one at 10 months (Case 1) and one at 3 months (Case 7). Five patients (Cases 1, 2, 3, 7, and 8) were treated by their dermatologist. Those who had only oral PV (Cases 4, 5, 6, 9, and 10) were managed in the dental office with topical corticosteroids combined with effective plaque control.

## 4 DISCUSSION

This study confirmed that there are no differences in clinical and laboratory findings regardless of the presence or absence of anti‐Dsg 3 antibodies in patients with mild to moderate oral PV. All of the clinical, cytological, histopathological, and DIF findings are consistent with PV, although only five of the 10 cases had a positive reaction to the Dsg 3 ELISA at the time of diagnosis (Tables 1 and 2). The Dsg 3 ELISA data do not always relate to the disease activity (Avgerinou et al., 2013; Belloni‐Fortina et al., 2009; Daneshpazhooh et al., 2007; Jamora et al., 2003; Khandpur et al., 2010; Koga et al., 2012; Sharma et al., 2006; Zagorodniuk et al., 2005). A few patients with PV had no detectable anti‐Dsg 3 antibody, even though they had active disease in the oral cavity (Avgerinou et al., 2013; Belloni‐Fortina et al., 2009; Daneshpazhooh et al., 2007; Jamora et al., 2003; Khandpur et al., 2010; Koga et al., 2012; Sharma et al., 2006; Zagorodniuk et al., 2005). However, these previous studies did not provide detailed information regarding the Dsg 3 ELISA negative PV findings. Therefore, it was previously unclear whether the disease expressions of Dsg 3 ELISA negative PV were identical to the Dsg 3 ELISA positive PV. The present study clearly shows that there are no differences in clinical, cytological, histopathological, or DIF findings, despite the presence or absence of anti‐Dsg 3 antibodies in patients with mild to moderate oral PV. The primary limitation of the present study is that the sample size is small. Future clinical research with a larger number of cases will greatly improve the strength of resulting conclusions. In a more sophisticated environment, it would be most beneficial to study all known or suspected potentially etiologic autoantigens.

The landmark concept of PV pathogenesis holds that autoantibodies against Dsg 3 are essential and sufficient for creating the epithelial acantholysis by disrupting the normal function of the desmosomes (Amagai et al., 2006; Stanley et al., 2001). As shown in the present study, however, the positive gingival Nikolsky's sign, the presence of acantholytic (Tzanck) cells in cytologic smears, acantholysis and a suprabasilar split in H&E‐stained sections, and intercellular deposits of (Ig)G in the DIF test were confirmed in all of the negative Dsg 3 ELISA cases, as well as the positive Dsg 3 ELISA cases (Tables 1 and 2). Moreover, the Dsg 3 ELISA in the follow‐up period increased and subsequently became positive in three cases (Cases 6–8) in which negative Dsg 3 ELISA was found at the time of diagnosis (Table 3). These results are inconsistent with the existing theory that the autoantibody against Dsg 3 is the cause of acantholysis in the oral mucosa. Specifically, the acantholysis had already occurred before the detection of Dsg 3 by ELISA. Some authorities postulate that the anti‐Dsg antibodies detected by ELISA are the “witnesses of disease” rather than the “cause” of PV acantholysis (Amagai et al., 2006). The data showed in the present study do not exclude this hypothesis from the pathogenesis of PV.

It is conceivable that if the total amount of anti‐Dsg3 antibodies was very low in a PV case, most antibodies would bind to the mucous membrane and skin, and there were few anti‐Dsg3 antibodies in circulation. However, the sensitivity of the Dsg 3 ELISA is very high, and false negatives are rare (Amagai et al., 1999). Therefore, it is unlikely that patients have Dsg 3 antibodies at levels that can not be detected by ELISA testing. Currently, there is unequivocal evidence that Dsg 3 is only one of the many targets of PV autoimmunity (Ahmed et al., 2016; Amagai et al., 2006; Amber et al., 2018; Cirillo et al., 2012; Di Zenzo et al., 2017; Grando, 2012; Sardana et al., 2013). Therefore, the PV patients in whom Dsg3 was not detected by ELISA may have other non‐Dsg targets. If non‐Dsg antibodies alone were responsible for acantholysis in some cases of PV, autoantibodies to the various antigens on the keratinocyte surfacing detected by DIF but negative by Dsgs ELISA testing (Ahmed et al., 2016). This could apply to the two cases (Cases 9 and 10) in which Dsg 3 ELISA was not detected even after for a long follow‐up period (Table 3).

Anti‐Dsg 3 antibody was first considered as the principal cause of separation of desmosomes because the binding of anti‐Dsg 3 antibody to Dsg 3 caused direct inhibition of desmosome function (the monopathogenic theory; Ahmed et al., 2016; Stanley & Amagai, 2006). The main postulate of this theory is that antibody‐dependent disabling of Dsgs‐mediated cell–cell attachment is sufficient to disrupt epithelial integrity (Ahmed et al., 2016; Stanley & Amagai, 2006). However, the electron microscopic studies of PV lesions demonstrate that the loss of cell–cell adhesion seems to occur first in the interdesmosomal areas where there are no desmosomes (Diercks, Pas, & Jonkman, 2009). The desmosomal separation may occur as the final rather than the primary or causative event in PV acantholysis (Diercks et al., 2009). Current evidence favors the idea that the pathogenic non‐Dsg autoantibodies initiate signal transduction by acting on receptors on keratinocyte surfaces, resulting in acantholysis through a more complex process (the multipathogenic theory; Ahmed et al., 2016; Bystryn & Grando, 2006). This theory explains acantholysis through the “multiple hit” hypothesis. The main postulate of this theory is that non‐Dsg antibodies that can induce one or more of the PV‐relevant keratinocytes changes, such as cell shrinkage, cell–cell detachment, and/or proapoptotic signaling (Ahmed et al., 2016; Bystryn & Grando, 2006). The damaged keratinocytes caused by cell shrinkage may act as antigen‐presenting cells and subsequently produce the anti‐Dsg 3 antibody as a scavenger antibody (Grando, 2012). This may explain why elevation of the Dsg 3 ELISA value can occur after acantholysis, as shown in the present study (Table 3).

Koga et al. (2012) reported five Japanese patients with PV who had oral lesions but no evidence of Dsg 3 ELISA in the serum. All the patients had a positive reaction for Dsg 1 ELISA. Other clinical, histopathological, and DIF findings were consistent with PV rather than PF. Koga's study suggests that antigenic diversity of anti‐Dsg 1 antibodies may be one of the causes of acantholysis in the oral mucosa. In the present study, only two patients had a positive reaction in Dsg 1 ELISA measurement (Table 2). One patient (Case 7) who had oral lesions but no evidence of Dsg 3 ELISA had a positive reaction in Dsg 1 ELISA. The authors believe that this patient does not have PF because acantholysis was confirmed just above the basal cell layer in the H&E‐stained section. This observation is a histopathologic hallmark of PV patients.

This study reported that the Dsg 3 ELISA was negative in some PV patients presenting with desquamative gingivitis. Therefore, PV acantholysis may precede the elevation of circulating anti‐Dsg 3 antibody levels. Non‐Dsg antibodies alone may be responsible for acantholysis in some cases of PV. ELISA is a sensitive and easy‐to‐use test for the diagnosis of pemphigus; however, diagnosing PV should combine ELISA with a comprehensive approach, using clinical, histopathological, and immunofluorescent criteria.

## FUNDING INFORMATION

This study was supported by a Grant‐in‐Aid for Young Researcher Scientists, Nihon University School of Dentistry at Matsudo (2015).

## CONFLICT OF INTEREST

The authors report no conflicts of interest related to this study.
